# A Method of Forming Sockets for Gum Teeth on Plates

**Published:** 1853-04

**Authors:** J. Taft

**Affiliations:** Xenia, O


### *A Method of Forming Sockets for Gum Teeth on Plates.*

By Dr. J. TAFT, Xenia, O.

All are familiar with the ordinary method of wiring or rimming, the outer edges of plates; the heel of the tooth rests upon it, and is thus strengthened. Plates so formed are stronger; and a better edge is presented for a contact with the soft parts.

The operation I am about to describe is mainly an extension of these advantages. I have made an effort to accomplish the same end in three different ways, each of which I will describe. In the first effort I used gold foil, No. 30, folded it into a strip of eight thicknesses, and about two and a half lines in width. After the teeth were all ground on, and retained in place, by adhesive wax, I took the strip thus prepared, and applied it to the outer edge of the plate making it to overlop the heels of the teeth, a little more than a line, fastening it to the plate with iron wire clasps; then with a burnisher rub the upper edge of the strip closely to each tooth; after it is made to fit all the teeth on the piece perfectly; then warm the plate and remove the teeth and adhesive wax carefully, allowing the strip to remain unmoved. Then solder the strip to the plate, also flow on enough solder to unite all the plies of which the strip is formed; care should be taken that too much solder does not flow into the sockets formed by the strip, also that the edge of the strip is not melted off.

In consequence of the difficulties liable to occur in this operation, I experimented still farther. Instead of using the foil, I took fine gold, and rolled it down to just that thickness, that I could fit it about the heels of the teeth with the burnisher; which was five or six times as thick as the foil. It required two or three plies of this to form the sockets; they were well formed. The principal difficulty was that there was more solder used than is desirable.

To remedy that difficulty, I adopted another method of procedure, and it I think the best. After the teeth are ground on, and all in place by the wax, cast plaster about the gums of the teeth, and the projection of the plate; cast it so that it will separate at the median line, that it may be drawn readily from the teeth and plate, we then have two plaster counter-models for each piece, if full arches, from these get up plaster models, and then a model and counter model, in metal. Then take strips of plate, No. 28 to 30, and wedge them in those casts: now when applied to their respective places on the pieces they will fit perfectly if the manipulation has been correct.

The strips are now retained in place by wire clasps, and the teeth and wax removed as before. Then that part of the plate from which the teeth and wax have been removed should be covered with plaster, permitting it to flow into and fill up the spaces between the main plate and strip, this prevents the solder from intruding into that space. The strip is now perfectly united to the plate by soldering, the solder should not be run upon the outside of the strip, and it will be much easier finished.

We now have a perfect socket to receive the heel of each tooth and fit it nicely. The depth of the socket should be about a line and a half, or sufficient to retain the heels of the

The teeth can now be replaced, backed, soldered, and finished in the usual manner. It gives a neat appearance to the work, adds very much to the strength of a piece, the plate will be much stiffer; even a narrow lower plate thus fitted up, can scarcely be warped by the hands.

It adds much to the strength of the teeth; the rivets do not sustain the force brought on teeth in mastication, but it comes upon the sockets, the rivets only serving to keep the teeth in place. If the sockets are properly formed, it will be impossible for the teeth to be bent inward, but must retain their position unless the gum is broken off.

By this method plates may be much lighter than they should otherwise be, and yet be stiffer. Plates are not so liable to warp in soldering on the teeth when thus made; indeed I think it almost impossible to spring.

